# The Edible Brown Seaweed *Ecklonia cava* Reduces Hypersensitivity in Postoperative and Neuropathic Pain Models in Rats

**DOI:** 10.3390/molecules19067669

**Published:** 2014-06-10

**Authors:** Jae Goo Kim, Dong Wook Lim, Suengmok Cho, Daeseok Han, Yun Tai Kim

**Affiliations:** 1 Food Resource Research Center, Korea Food Research Institute, Seongnam 463-746, Korea; E-Mails: yadj2654@naver.com (J.G.K.); neodw4015@kfri.re.kr (D.W.L.); smcho@kfri.re.kr (S.C.); imissu@kfri.re.kr (D.H.); 2 Research Group of Food Functionality, Korea Food Research Institute, Seongnam 463-746, Korea; 3 Korea University of Science and Technology, Daejeon 305-350, Korea

**Keywords:** edible seaweed, *Ecklonia cava*, analgesic activity, pain, hyperalgesia, allodynia, ultrasonic vocalization

## Abstract

The current study was designed to investigate whether edible brown seaweed *Ecklonia cava* extracts exhibits analgesic effects in plantar incision and spared nerve injury (SNI) rats. To evaluate pain-related behavior, we performed the mechanical withdrawal threshold (MWT) and thermal hypersensitivity tests measured by von Frey filaments and a hot/cold plate analgesia meter. Pain-related behavior was also determined through analysis of ultrasonic vocalization. The results of experiments showed MWT values of the group that was treated with *E. cava* extracts by 300 mg/kg significantly increased; on the contrary, number of ultrasonic distress vocalization of the treated group was reduced at 6 h and 24 h after plantar incision operation (62.8%, *p* < 0.05). Moreover, *E. cava* 300 mg/kg treated group increased the paw withdrawal latency in hot-and cold-plate tests in the plantar incision rats. After 15 days of continuous treatment with *E. cava* extracts at 300 mg/kg, the treated group showed significantly alleviated SNI-induced hypersensitivity response by MWT compared with the control group. In conclusion, these results suggest that *E. cava* extracts have potential analgesic effects in the case of postoperative pain and neuropathic pain in rats.

## 1. Introduction

Pain management remains a major clinical challenge, because there is not an appropriate understanding about the mechanisms causing and maintaining pain and effective treatments [1]. Therapeutic drugs for treating pain have limited effectiveness and safety [2]. Repetitive use of non-steroidal anti-inflammatory drugs (NSAIDs) may cause adverse effects such as gastrointestinal lesions or renal and liver failure [3]. Furthermore, current analgesic drug, even including the opioids, cannot make the pain easier in some painful conditions like neuropathic pain. [4]. Therefore, it is necessary to research new effective and safe analgesics among natural product-derived secondary metabolites.

Marine plants, and particularly the edible brown seaweed (*Ecklonia cava*), which is abundantly produced on Jeju Island in Korea, has been widely used in such as an ingredient for food, animal feed, and fertilizers [5]. In particular, in Korea, brown seaweeds are well known as a folk medicine administered to new mothers after birth [6]. *Ecklonia* species include various constituents such as phenols, carotenoids, and polysaccharides, reported to have antioxidant [7], antibacterial [8], anticoagulant [9], anti-diabetic [10], and anti-inflammatory [11] activities. Recently, our group found that administration of *E. cava* extracts and its phlorotannins-rich fractions induces sleep duration in pentobarbital-induced sleep tests in mice. It was also shown that this effect might be via modulation of benzodiazepine sites on Gamma-amino butyricacid type A (GABA_A_) receptors [12,13]. Also, variously modulated GABA_A_ receptors reduced the behavioral symptoms in animal models of experimental pain [14]. *E. cava* was therefore, considered a candidate for the effective treatment of pain-related disorders because of its rich phlorotannins content [15]. However, no studies have been made of the effect of *E. cava* extracts on the surgical incision of postoperative pain or neuropathic pain *in vivo* models.

T present study was designed to investigate whether *E. cava* extracts exhibits anti-nociceptive effects in the model of postoperative pain through plantar incision [16] and on the spared nerve injury (SNI) rat model of neuropathic pain [17]. To evaluate pain-related behavior, we studied the mechanical withdrawal threshold (MWT) as measured by von Frey filaments, and the pain-induced ultrasonic vocalizations (USVs) have been examined by ultrasonic microphones [18].

## 2. Results and Discussion

### 2.1. Effects of Ecklonia cava Extracts on Mechanical and Thermal Hypersensitivity Induced by Plantar Incision

**Figure 1:**
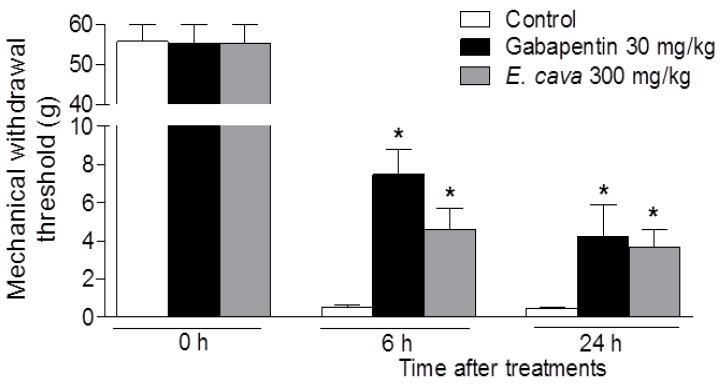
Effect of *E. cava* extracts on mechanical hypersensitivity induced by plantar incision in rats. Baseline assessment of animals, before surgery (day 0), showed no significant variation between groups. 6 h or 24 h after surgery, rats treated with *E.* *cava* extracts significantly attenuated hypersensitivity in response to von Frey stimulation of injured hind paw. Data are mean ± SEM (n = 7 per group). * *p* < 0.05, significant difference from the control group.

The analgesic activity of *E. cava* extracts was determined using the postoperative pain model in rats. Postoperative pain in humans can be mimicked by plantar incision in rats [16]. Incision of the plantar surface of the hind paw produced a significant reduction in the mechanical withdrawal threshold (MWT), as measured using the von Frey assay, and GABA agonist including gabapentin, effectively reverse incision-induced decreases in the MWT against mechanical hypersensitivity [19]. The plantar incision produced a marked mechanical hypersensitivity in the incised paw (paw withdrawal threshold diminished from 55.75 ± 4.25 g at baseline to 0.47 ± 0.01 g 24 h after plantar incision; *p* < 0.001). Administration of *E. cava* extracts (300 mg/kg, p.o.) significantly attenuated hypersensitivity in response to von Frey stimulation of the injured hind paw as evidenced by an increased MWT values as compared to control rats (3.68 ± 0.91 g *vs.* 0.47 ± 0.01 g, *p* < 0.05) 24 h after incision surgery (Figure 1).

**Figure 2:**
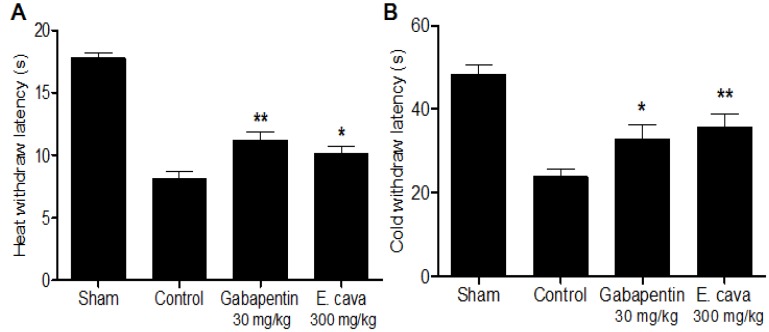
Effect of *E.* *cava* extracts on thermal hypersensitivity induced by plantar incision in rats. 24 h after surgery, rats treated with *E.* *cava* extracts significantly attenuated (**A**) heat and (**B**) cold hypersensitivity in the stimulation of injured hind paw. Data are mean ± SEM (n = 7 per group). ** *p* < 0.01 and * *p* < 0.05, significant difference from the control group.

Also, the plantar incision surgery induced a decreased of the paw withdrawal latency during a thermal stimulus (heat and cold) in comparison to non-injury rats [20,21]. In our results incisional surgery produced immediate (24 h after surgery) thermal hypersensitivity in the injured hind paws. Gabapentin significantly blocked thermal hypersensitivity in the injured hind paws. Administration of *E.* *cava* extracts (300 mg/kg, p.o.) significantly attenuated hypersensitivity in response to thermal stimulus of the injured hind paw as evidenced by an increased withdrawal latency values by 24.1% and 50.2%, for the heat and cold assessments, respectively (Figure 2).

### 2.2. Effects of E. cava Extracts on Ultrasonic Vocalizations Induced by Plantar Incision

The anti-nociceptive activity of *E. cava* extracts was also determined by the pain-induced ultrasonic vocalizations (USVs) method using ultrasonic microphones. Adult rats produce two distinct types of USVs that appear to reflect the caller’s emotional state: either positive (a high-pitched and short ~50 kHz USVs) or negative state (a low-pitched and longer ~27 kHz USV) [22]. In particular, 22–27 kHz USVs have been suggested as being a measure of affective shifts in rats [23] and have been used in a variety of unconditioned models such as pain, anxiety and stress-related models [24,25].

**Figure 3:**
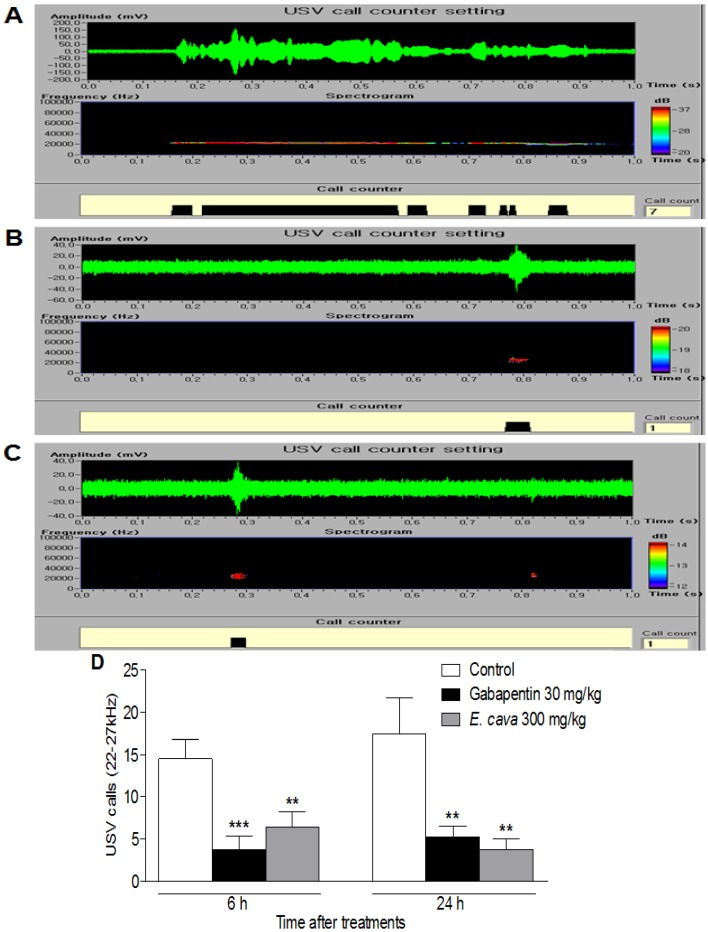
Effect of *E.* *cava* extracts on USVs induced by plantar incision in rats. The sonograms of USVs in (**A**) control, (**B**) gabapentin and (**C**) *E.* *cava* extracts treated rats. (**D**) A significant difference in 22–27 kHz USV calls was observed between the *E.* *cava* extracts treated group and the control group. Data are mean ± SEM (n = 7 per group). ** *p* < 0.01 and *** *p* < 0.001, significant difference from the control group.

Pain-induced USVs have been examined by ultrasonic microphones, because vocalization is not only an objective but also a quantifiable value in the rats [26]. After 6 h or 24 h after plantar incision, the control group emitted 22–27 kHz USV calls, in pain-related behaviors [27]. The group treated with 30 mg/kg of gabapentin showed significantly reduced 22–27 kHz USV calls compared with the control group, showing its anti-nociceptive effects in rats. *E. cava* extracts also reduced 22–27 kHz USV calls; a significant reduction was observed after the administration of *E. cava* extracts at 300 mg/kg (62.8%, *p* < 0.01 *vs.* control) (Figure 3). According to our findings, compared to the experimental group treated with gabapentin, the *E. cava* extracts might have anti-nociceptive effects on plantar incision postoperative pain in rats.

### 2.3. Effects of E. Cava Extracts on Mechanical Hypersensitivity Induced by Spared Nerve Injury

**Figure 4:**
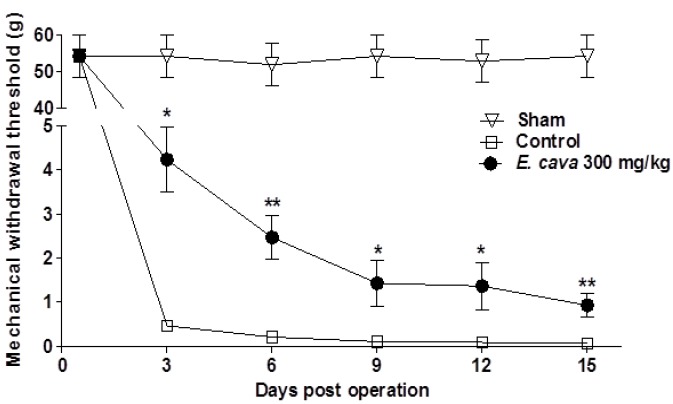
Effect of *E. cava* extracts on SNI rat model of neuropathic pain. Administration of *E. cava* extracts (300 mg/kg, p.o) significantly attenuated hypersensitivity in response to von Frey stimulation of hind paw from 3 to 15 day after treatment. Data are mean ± SEM (n = 7 per group). ** *p* < 0.01, and * *p* < 0.05 significant difference from control group.

In this study we evaluated potential efficacy of *E. cava* extracts in rat model of the spared nerve injury (SNI) with regard to neuropathic pain. SNI mimics the symptoms of chronic nerve compression in human [28]. At baseline (day 0), there are no significant changes between the *E. cava* extracts (300 mg/kg) treated group and SNI-control group. Animals began to show hypersensitivity response to von Frey stimulation after 3 day of operation on the process of experiments. The frequency of withdrawals in SNI-control group significantly reached from on 54.33 ± 5.67 g day 0 to on 0.06 ± 0.02 g day 15 after SNI-operation. Administration of *E.* *cava* extracts significantly attenuated hypersensitivity in response to von Frey stimulation of hind paw evidenced by an increased MWT values as compared to SNI-control rats from 3 to 15 day after treatment (** *p* < 0.01) (Figure 4). γ-Aminobutyric acid type A (GABA_A_) receptors are the major transduced of fast inhibitory neurotransmission in the central nerve system [29]. A loss of GABA_A_ receptor-mediated inhibitory neurotransmission circuits is an important contributor for the development of pain and one of a number of key mechanisms contributing to behavioral hypersensitivity in animal models [30,31]. An alternative treatment for pain has emerged with the development of gabapentin (neurontin), a structural analogue of GABA, which has recently been shown to reduce the hypersensitivity associated with animal models of neuropathic pain [32,33]. Moreover, gabapentin has been shown to be effective in clinical conditions of neuropathic pain, which are resistant to standard analgesics [34]. Taken together, it might be hypothesized that *E. cava* extracts attenuated the behavioral symptom of neuropathic pain in rats.

## 3. Experimental

### 3.1. Preparation of E. cava Extracts

Dried *E. cava* was purchased from S & D Co., Ltd. (Chungcheongbuk-do, Korea). Whole dried *E. cava* (300 g) was extracted with 70% ethanol (3,000 mL) for 4 h at 80 °C in a reflux apparatus. The process was repeated twice, and the extracts were filtered through membrane filters (0.45 µm; Millipore, Billerica, MA, USA). The samples were lyophilized to yield a dark yellow powder. The yield of *E. cava* extracts was 19.46%.

### 3.2. Animals and Treatments

Male Sprague-Dawley (SD) rats (160–200 g) were purchased from Samtako (Gyeonggi-do, Korea). Animals were housed at two rats per cage in an air-conditioned room at 23 ± 1 °C, 55%–60% relative humidity, and a 12 h light/dark cycle (07:00 lights on, 19:00 lights off), and were given a laboratory regular rodent diet. After acclimatization for 1 week, 8-week-old male SD rats were anesthetized with 2% of isoflurane and pain-related surgeries were operated. After plantar incision operation, rats were divided into three following treatment groups: (1) control + vehicle, (2) control + gabapentin (30 mg/kg, i.p.), and (3) control + *E. cava* extracts (300 mg/kg, p.o.). *E. cava* extracts and gabapentin (Sigma, MO, USA) were dissolved in distilled water for oral administration at the desired doses in a volume of 5 mL/kg. The sample treated groups were oral administrated *E. cava* extracts or gabapentin at 1 h after plantar incision operation. After spared nerve injury (SNI) operation, rats were divided into two following treatment groups: (1) SNI-control + vehicle, and (2) SNI-control + *E. cava* extracts (300 mg/kg). *E. cava* extracts was given by p.o. route, immediately following surgery, once a day which continued for 15 consecutive days. All animal experiments were carried out according to the guidelines of the Korea Food Research Institutional Animal Care and Use Committee (KFRI-M-12024).

### 3.3. Plantar Incision of Postoperative Pain Rat Model

Surgery was performed as previously described [16], with minor modifications. Briefly, rats were anaesthetized with 2% isofluorane and, a 1 cm longitudinal incision was made with scalpel, through skin and fascia of the plantar aspect of the paw, starting 0.5 cm from the proximal edge of the heel and extending toward the toes. The plantar is muscle was elevated and incised longitudinally. Following haemostasis with gentle pressure, the skin was opposed with two single interrupted sutures using polyamide monofilaments. The animals allowed recovering in their home cages.

### 3.4. Spared Nerve Injury (SNI) of Neuropathic Pain Rat Model

The surgical procedure was performed as described previously with some modifications [35]. The SNI procedure comprised an axotomy and ligation of the tibial and common peroneal nerves leaving the sural nerve intact. The common peroneal and the tibial nerves were tight-ligated with 5.0 silk and sectioned distal to the ligation, removing 2 ± 4 mm of the distal nerve stump. Great care was taken to avoid any contact with or stretching of the intact sural nerve. The skin was opposed with two single interrupted sutures using polyamide monofilaments.

### 3.5. Mechanical Withdrawal Threshold (MWT) Analysis

Animals were placed on an elevated wire grid and the plantar surface of the paw stimulated with a series of ascending force von Frey monofilaments (Stoelting, Chicago, IL, USA). The threshold was taken as the lowest force that evoked a brisk withdrawal response to one of three repetitive stimuli. To determine the time course of hypersensitivity, a baseline measurement was made prior to surgery, and then again at 6 and 24 h post-surgery for postoperative pain, 3, 6, 9, 12, and 15 days post-surgery for Neuropathic Pain.

### 3.6. Heat and Cold Withdrawal Latencies Analysis

To assess thermal hypersensitivity to heat and cold stimulus in rats, the hot/cold plate analgesia meter (IITC Life Science, Woodland Hills, CA, USA) was used according to a minor modification of the described methods [36]. The rats were placed on the aluminum plate maintained at 25–28 °C in the chamber of the device for acclimatization. To analyze thermal hypersensitivity rats were placed in the hot plate (50 °C) or in the cold plate (0 °C). The latency to licking the hind paws or jumping was measured for each rat. The cut-off latency for hot plate test was 60 s and for cold plate test was 120 s. The thermal hypersensitivity behavior was tested 24 h after plantar incision.

### 3.7. Ultrasonic Vocalization (USVs) Analysis

After induction of the plantar incision of postoperative pain, the category of 22–27 kHz USVs emitted by adult rats was monitored and scored for 10 min using Sonotrack ultrasonic microphones (Metris B.V., KA Hoofddorp, The Netherlands) placed at a distance of 25–30 cm from the heads of the animals. The rats emitted ‘calls (number of USVs)’ that were counted using the Sonotrack 2.2.1 software.

### 3.8. Statistical Analysis

Data analyses were performed using one-way analysis of variance (ANOVA), followed by Tukey’s post hoc test, using Prism 5 (GraphPad Software, Inc., San Diego, CA, USA) for multi-group comparisons. All data are presented as the mean ± standard error (SEM). Significance was set at *p* < 0.05.

## 4. Conclusions

In conclusion, the analgesic effect of *E. cava* extracts led to a reduction in the number of ultrasonic distress vocalization by plantar incision of postoperative pain in rats, and to a decrease in hypersensitivity in response to von Frey stimulation of hind paw evidenced by a decreased mechanical withdrawal threshold (MWT) in the spared nerve injury (SNI) of neuropathic pain rats. These results suggest that *E. cava* extracts could be useful on the treatment of postoperative and neuropathic pains, but more pharmacological and toxicological investigations are needed for finding the exact mechanism of action and for safety evaluation.
